# Oxidative stress and skeletal muscle dysfunction are present in healthy smokers

**DOI:** 10.1590/1414-431X20165512

**Published:** 2016-10-24

**Authors:** C.D.C. Neves, A.C.R. Lacerda, V.K.S. Lage, L.P. Lima, R. Tossige-Gomes, S.F. Fonseca, E. Rocha-Vieira, M.M. Teixeira, V.A. Mendonça

**Affiliations:** 1 Programa Multicêntrico de Pós-Graduação em Ciências Fisiológicas, Universidade Federal dos Vales do Jequitinhonha e Mucuri, Sociedade Brasileira de Fisiologia, Diamantina, MG, Brasil; 2 Laboratório de Inflamação e Metabolismo, Universidade Federal dos Vales do Jequitinhonha e Mucuri, Diamantina, MG, Brasil; 3 Laboratório de Imunofarmacologia, Universidade Federal de Minas Gerais, Belo Horizonte, MG, Brasil

**Keywords:** Inflammation, Muscle endurance, Redox balance, Smoking, Tobacco cigarette

## Abstract

Chronic exposure to cigarette smoke seems to be related to an increase of pro-inflammatory cytokines, oxidative stress and changes in muscular and physical performances of healthy smokers. However, these parameters have not yet been evaluated simultaneously in previous studies. The participants of this study were healthy males divided into two groups: smokers (n=20) and non-smokers (n=20). Inflammation was evaluated by measuring plasma levels of the cytokines IL-10, IL-6 e TNF-α, and of the soluble receptors sTNFR1 and sTNFR2. Oxidative stress was evaluated by determination of thiobarbituric acid reactive substances (TBARS) plasma levels, total antioxidant capacity of plasma and erythrocytes activity of the antioxidant enzymes superoxide dismutase (SOD) and catalase. Muscular performance was evaluated by measuring the peak torque of knee flexors and extensors, and by determining the total work of the knee extensors. Physical performance was assessed by measuring the peak oxygen uptake (VO_2_ peak), the maximum heart rate (HRmax) and the walking distance in the shuttle walking test. Smokers showed an increase in the levels of the sTNFR1 and TBARS and a decrease in the total antioxidant capacity of plasma, in the catalase activity and in the total work (P<0.05). IL-6, IL-10, sTNFR2, SOD, peak torque, VO_2_ peak, HRmax and walking distance were similar between groups. Smokers presented increased oxidative stress and skeletal muscle dysfunction, demonstrating that the changes in molecular and muscular parameters occur simultaneously in healthy smokers.

## Introduction

Considered as a pandemic by the World Health Organization, smoking is the main preventable cause of death worldwide (1). Currently, about six million people die each year as a consequence of diseases caused by tobacco, and it is estimated that, by 2030, the number of deaths could increase to ten million people per year if the usage trend persists (1).

The development of major tobacco-related diseases is due mainly to chronic exposition to toxic components of the cigarette smoke, generated from the combustion of tobacco. Several lines of evidence suggest that the pathophysiology of the main tobacco-related diseases and many adverse systemic consequences of chronic smoking might be due to its effects on oxidative stress and on inflammation (2,3). Oxidative stress and inflammation have also been identified as potential etiological factors of skeletal muscle dysfunction (4,5), the main extra-pulmonary manifestation of chronic obstructive pulmonary disease (COPD), for which smoking is the main risk factor (6). Skeletal muscle dysfunction (loss of strength and/or muscle endurance) has a considerable impact on the exercise tolerance of the patients (7) that may begin at the same time as the lung abnormalities, or may even precede them (4,8).

Thus, some studies have evaluated the effects of smoking on oxidative-inflammatory response and on the muscular system of healthy smokers, who have normal lung function and absence of tobacco-related diseases. Chronic smoking in healthy smokers has been shown to be associated with higher levels of the pro-inflammatory cytokine TNF-α (9,10) and the products of lipid peroxidation (11,12). Moreover, the reduction of muscle endurance (13,14) and physical performance (15) has been reported, suggesting that, as in patients with COPD, the chronic exposition to cigarette smoke seems be related to inflammation, oxidative stress (9,10,16) and skeletal muscle dysfunction (8,13,14) in healthy smokers. However, the knowledge about the effects of smoking on healthy smokers is still uncertain because most studies are performed on subjects with associated diseases, hospitalized patients and old smokers that have been smoking for a long time, making it difficult to determine the effects of smoking on healthy adult smokers.

Although there are studies that point to molecular or musculoskeletal changes in healthy smokers, it is worth mentioning that simultaneous assessment of inflammatory-oxidative and muscular parameters has not been performed in previous studies with this population. These assessments would facilitate the identification of early changes caused by smoking that are similar to those observed in patients with COPD. Therefore, this study aimed to evaluate inflammatory and oxidative stress indicators and the muscular and physical performance of healthy smokers.

## Material and Methods

### Study subjects

This study involved healthy male participants, aged 18-45 years, that were recruited by personal invitation at home visits and health centers in the local community. The smoking group was composed of twenty smokers, defined as those who have smoked at least 100 cigarettes and were smokers at the time that the study was conducted (17). The control group was composed of twenty non-smokers.

To be included in the study, the subjects had to meet the following criteria: normal lung function, self-report of no current acute or chronic diseases; be eutrophic according to body mass index (BMI between 18.5–24.9 kg/m^2^), not currently using anti-inflammatory medications and self-reported absence of cough, infection, fever and flu in the month prior to the assessments. In addition to the above noted criteria, control subjects could not be passive smokers, and smokers must have been smoking cigarettes manufactured with a filter. This study followed the declaration of Helsinki. The Ethics and Research Committee of the Universidade Federal dos Vales do Jequitinhonha e Mucuri, Brazil, approved this study (protocol #003/12). All participants gave written, informed consent.

### Clinical assessment and lung function

To assess the body composition, weight and height were measured on an anthropometric mechanical scale, BMI was calculated as the weight divided by the height squared, and body fat percentage was estimated by measuring skinfold thickness using a plicometer.

Lung function was measured using a digital spirometer (PonyFX^®^, Cosmed, Italy). The forced expiratory volume in 1 second (FEV_1_), forced vital capacity (FVC) and FEV_1_/FVC were calculated in accordance with the American Thoracic Society and the European Respiratory Society (18). The percentages of predicted spirometry values were calculated from published Brazilian population data (19).

### Smoking history and level of exposure to cigarette smoke

The smoking history of the smoker subjects was determined through self-report of the number of pack-years, calculated as the number of smoked cigarettes per day/20 and multiplied by the number of years of smoking (17).

To assess the level of exposure to cigarette smoke and the amount of nicotine absorbed, blood collection was performed (described below) to measure the levels of serum cotinine. The blood was collected in tubes without anticoagulant and immediately transported to a private laboratory that performed the analysis by the chemiluminescence method. A level of cotinine above 25 ng/mL was considered as the reference value for smokers.

### Analysis of blood

For the analysis of cytokines, soluble receptors, oxidative stress, and additionally of cotinine in the smokers, blood was collected between 6:00 and 8:00 am. The blood was collected aseptically by puncturing the median cubital vein. The volunteer fasted and abstained from cigarettes in the 8–12 h prior to collection.

The plasma levels of cytokines IL-10, IL-6 and TNF-α and the soluble receptors of TNF-α, sTNFR1 and sTNFR2 were measured by ELISA kits (DuoSet^®^, R&D Systems, USA) according to the manufacturer's instructions. The limits of detection were of 10 pg/mL for IL-6, IL-10, sTNFR1, sTNFR2 and 5 pg/mL for TNF-α.

Oxidative stress was evaluated by determining plasma levels of thiobarbituric acid reactive substances (TBARS) (20), total antioxidant capacity of plasma (21) and erythrocyte activity of the antioxidant enzymes superoxide dismutase (SOD) (22,23) and catalase (24), according to previously published methods.

### Assessment of muscular and physical performance

Muscular performance of the dominant leg was evaluated by measurements of the peak torque and total work with the use an isokinetic dynamometer (Biodex Medical Systems Inc.^®^, USA). Isokinetic knee flexor and extensor testing was performed in a concentric–concentric regime. Tests consisted of five maximum repetitions of isokinetic contractions at a speed of 60°·s^-1^ and thirty at a speed of 180°·s^-1^, separated by a resting period of 5 min. The variables analyzed were the peak torque of flexion and extension of the knee at a speed of 60°·s^-1^ (25) and the total work of extension of the knee at a speed of 180°·s^-1^ (26).

Physical performance was evaluated by cardiorespiratory fitness in the shuttle walking test (SWT). To perform the SWT, the participants were instructed to walk a distance of 10 m around a mark between two cones placed 0.5 m from each endpoint. The walking speed at which the participant should walk was dictated by a sound played from a CD that was originally generated by a microcomputer. Each minute, the walking speed increased by 0.17 m/s, with an initial speed of 0.5 m/s. The test was terminated when the volunteer was not able to maintain the required speed (more than 0.5 m from the cone), at the request of the volunteer, or because of some other reported symptom (dyspnea, dizziness, vertigo, angina). As suggested by the literature, we used a protocol of 15 levels to evaluate the maximum cardiorespiratory capacity of healthy participants (27). Before and after the test, the heart rate (HR, measured by a heart rate monitor), blood pressure (measured by a mercury sphygmomanometer cuff and a stethoscope) and rating of perceived exertion (RPE, Borg scale, range 6–20) were measured. Additionally, the laps were recorded to calculate the walking distance.

During the test, the exhaled gases were collected using a gas analyzer via the portable telemetry system (K4b2, Cosmed), and the oxygen uptake (VO_2_) and HR was monitored breath-by-breath. The data was filtered, the VO_2_ peak was defined as the highest value obtained from the arithmetic mean of the log intervals of 30 s, and the maximum HR (HRmax) was defined as the highest HR value recorded during the test. Predicted HRmax was calculated from the equation HRmax = 220 – age.

### Statistical analysis

The statistical analysis was performed using the statistical package GraphPad Prism 4 (GraphPad Software Inc.,^®^ USA). The normality of data was checked by the Shapiro-Wilk test, and the comparison of results of smokers and control subjects was performed through the independent *t-*test for parametric variables or the Mann-Whitney test for non-parametric variables. The calculation of sample size was based on quadriceps maximal voluntary contraction and levels of the SOD enzyme of the Barreiro et al. (8) study. An alpha error of 0.05 and a power of 0.8 was selected, and reached a sample size of 8 subjects per group. The level of statistical significance was P≤0.05.

## Results

The clinical characteristics and the spirometric data of smokers and control subjects, the smoking history and the level of exposure to cigarette smoke of the smokers are presented in Table 1. There was no statistical difference between the groups with respect to age, weight, height, BMI, and fat percentage. FEV_1_ and FVC were similar between the groups; however, smokers presented statistically lower values for the FEV_1_/FVC ratio. Expressive levels of serum cotinine (median of 142.5 ng/mL; 82.5% higher than the reference value) were observed in smokers.

**Table:**
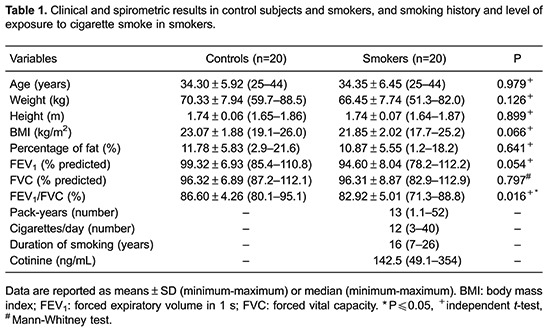

**Figure 1:**
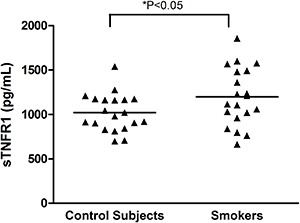
sTNFR1 concentration in control subjects and smokers. The independent *t*-test was used for statistical analyses.

Higher levels of sTNFR1 were found in the smoker group than in the control subjects; this fact indicated that this receptor performed an inflammatory role (Figure 1). The levels of sTNFR2 (P=0.378), IL-6 (P=0.074) and IL-10 (P=0.220) did not differ between smokers and control subjects. TNF-α was not detected in either group (data not shown).

**Figure 2:**
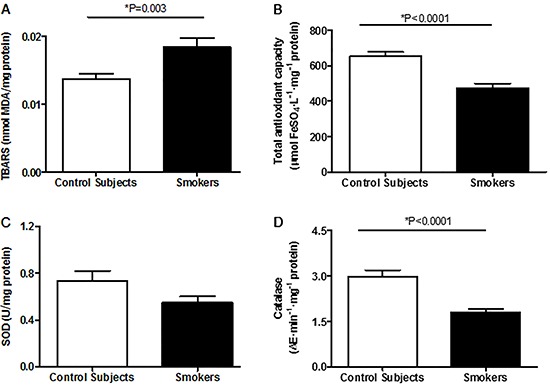
Oxidative stress results. TBARS: thiobarbituric acid reactive substances (*A*); total antioxidant capacity (*B*); SOD: superoxide dismutase (*C*); catalase (*D*). The independent *t*-test was used for statistical analyses.

In relation to oxidative stress, the plasma level of TBARS was statistically higher in smokers than in control subjects. The activity of the catalase enzyme of the erythrocytes and the total antioxidant capacity of plasma were statistically lower in smokers than in the control subjects. While not statistically significant, the activity of the SOD enzyme was lower in smokers than in the control subjects (Figure 2).

The results of muscular and physical performance are presented in Tables 2 and 3, respectively. The peak torques of flexion and extension of the knee at a speed of 60°·s^-1^ were similar between the groups, but the total work of extension of the knee at a speed of 180°·s^-1^ was statistically lower in smokers than in control subjects. Moreover, there was no significant difference in the VO_2_ peak, HR and walking distance between smokers and control subjects.

**Table:**
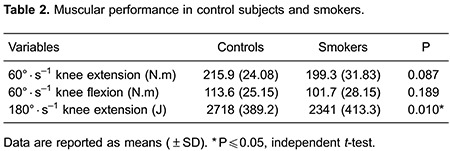

**Table:**
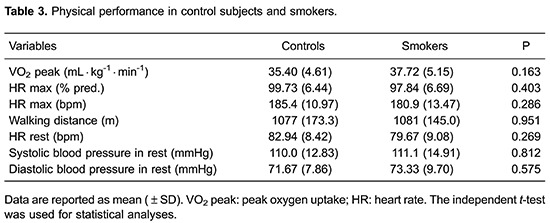

## Discussion

This study shows that healthy adult smokers suffer molecular and muscular changes, even without presenting tobacco-related diseases. Our findings are similar to previous studies that have not identified differences in levels of cytokines IL-6 and IL-10 in plasma (28), serum (29) and nasal lavage fluid (30) of healthy smokers compared to those of non-smokers. The absence of changes in these cytokines has been justified by the fact that young adults in the studies tend to have smaller changes in systemic levels of the cytokines than old subjects, which had more cigarette smoking time. Further, it has been suggested that the increased production of pro-inflammatory cytokines originates in the lung, as observed by an increase in the number of macrophages and the IL-6 and TNF-α concentrations in bronchoalveolar lavage, and the magnitude of the systemic inflammatory response is associated with the number of toxic particles phagocytized by macrophages (31). Thus, the greater the exposure to toxic components from the tobacco, the higher the concentration of these cytokines in peripheral blood. Therefore, the amount of exposure to tobacco in adult smokers in the present study may not have been enough to cause changes in the systemic levels of the IL-6 and IL-10 cytokines.

Although TNF-α was not detected in our subjects, probably because of the low sensitivity of the kit used for analysis, previous studies reported increased levels of TNF-α in the serum of adult smokers (9,10). The biological activity of TNF-α is mediated by two membrane receptors TNFR1 and TNFR2 that have different effects (32). The TNFR1 is associated with a pro-inflammatory response and apoptosis, and the TNFR2 is related to tissue repair and angiogenesis. Both receptors are released in their soluble form (sTNFR1 e sTNFR2) by proteolytic cleavage of forms associated with the surface membrane and play an important role in the regulation of the activity of TNF-α (33). Plasma detection of TNF-α is difficult because of its very low concentrations. Therefore, it has been suggested that its soluble receptors should be measured, because their levels in plasma are a consequence of exposure to TNF-α (32). Moreover, the presence of its soluble receptors is significantly delayed, because TNF-α have a shorter half-life in plasma, suggesting that sTNFRs could be more sensitive markers of TNF-α system activation (9). Therefore, it is believed that the increase of sTNFR1 in smokers is mainly due to a greater exposure to TNF-α, and, thus, to its pro-inflammatory effects.

Additionally, oxidative stress was observed in smokers as a result of an increase of oxidative damage in lipids, associated with a reduction in the antioxidant concentrations. An increase in TBARS levels has been reported in adult and elderly smokers. Aula and Qadir (11) and Nowak et al. (12) reported an increase of TBARS in serum and exhaled breath condensate of adult smokers that smoked a mean of 10–17 cigarettes per day. These findings show that the overload of oxidants in smokers, produced by the larger number of free radicals and oxidant molecules present in cigarette smoke (34), is associated with increased lipid peroxidation (12), which seems to be accompanied by an insufficient antioxidant defense (16,35) that may cause cell damage. This fact can be confirmed by the present data, as evidenced by the significant reduction in the total antioxidant capacity of plasma, in the catalase enzyme activity and, although non-significant, the lower activity of SOD enzyme in smokers.

In addition to molecular changes, healthy smokers exhibited lower total work of knee extensors, which demonstrated a possible skeletal muscle dysfunction (4,14). The muscle's total work is the action of strength over a specific distance, i.e, the action of torque during the range of motion. If this value is low, it may suggest that, clinically, the muscle function has changed and that the energy expended during a range of motion is not suitable or has a muscular deficit (36). In addition, the peak torque measures were found to be similar among the study's subjects. These results are in agreement with previous studies that showed that maximum peak torque (strength) of knee extensors was similar between smokers and non-smokers (13,14,37) and that only the endurance of these muscles was reduced in smokers (13,14). Histological and immunohistochemical studies on smokers' muscles have related these findings to changes in the composition of muscle fiber type, with a lower percentage and diameter of type I fibers and a higher percentage of type IIB fibers (37). The reduction in the supply of oxygen to muscles was reported to be the result of the high affinity of hemoglobin for carbon monoxide present in cigarette smoke (38), to the decline of the mitochondrial function because of the lower activity of cytochrome c oxidase (37), and to oxidative stress (8).

Unlike muscular performance, the physical performance of the smokers, based on the VO_2_ peak, maximum HR and walking distance were not different from those of the control subjects. The absence of chronic smoking influence on cardiorespiratory fitness suggests, in part, that the changes in the cardiovascular and respiratory systems are not sufficient to impact aerobic capacity. The reduction of cardiorespiratory fitness in adult smokers has been linked to a higher HR both at rest and during exercise (15) because of the increase in sympathetic activity caused by the action of nicotine on cardiac sympathetic nerves and high levels of plasma catecholamine (39). The resting HR, maximum HR and systolic and diastolic blood pressures at rest were similar among subjects of the present study, suggesting an absence of changes in autonomic cardiac control that could affect the cardiorespiratory fitness. Furthermore, as with inflammatory markers, the cardiorespiratory fitness also could be influenced by cigarette smoking. Strand et al. (40) observed that only middle-age male smokers that had smoked more than 15 pack-years presented a significant reduction in physical performance compared to non-smokers. Thus, it is believed that the amount of cigarette exposure by the subjects in this study, in spite of inducing changes in oxidative stress and muscular performance, was still not sufficient to induce significant changes in the inflammatory profile and cardiorespiratory fitness of the healthy smokers.

Confirming previous studies (5,8), this study demonstrated that skeletal muscle dysfunction can precede lung abnormalities in healthy smokers. In addition, our data demonstrated that simultaneous changes in molecular and muscular parameters occur in healthy smokers, similar to those observed in patients with COPD.

In this way, our results demonstrate that, although the inflammatory response and physical performance seemed unaffected in healthy smokers, chronic use of tobacco triggered molecular changes related to oxidative stress, as well as muscular changes related to skeletal muscle dysfunction.
